# The Effect of Surface Texture on Lubricated Fretting

**DOI:** 10.3390/ma13214886

**Published:** 2020-10-30

**Authors:** Agnieszka Lenart, Pawel Pawlus, Andrzej Dzierwa, Slawomir Wos, Rafal Reizer

**Affiliations:** 1 Engine Maintenance Europe Aero, Jasionka 954, 36-002 Jasionka, Poland; agnieszka.lenart@eme-aero.com; 2 Faculty of Mechanical Engineering and Aeronautics, Rzeszow University of Technology, Powstancow Warszawy 8 Street, 35-959 Rzeszow, Poland; adzierwa@prz.edu.pl (A.D.); wosslawomir@prz.edu.pl (S.W.); 3 College of Natural Sciences, University of Rzeszow, Pigonia Street 1, 35-310 Rzeszow, Poland; rreizer@ur.edu.pl

**Keywords:** lubricated fretting, gross slip regime, coefficient of friction, wear

## Abstract

Experiments were conducted using an Optimol SRV5 tester in lubricated friction conditions. Steel balls from 100Cr6 material of 60 HRC hardness were placed in contact with 42CrMo4 steel discs of 47 HRC hardness and diversified surface textures. Tests were carried out at a 25–40% relative humidity. The ball diameter was 10 mm, the amplitude of oscillations was set to 0.1 mm, and the frequency was set to 80 Hz. Tests were performed at smaller (45 N) and higher (100 N) normal loads and at smaller (30 °C) and higher (90 °C) temperatures. During each test, the normal load and temperature were kept constant. We found that the disc surface texture had significant effects on the friction and wear under lubricated conditions. When a lower normal load was applied, the coefficient of friction and wear volumes were smaller for bigger disc surface heights. However, for a larger normal load a higher roughness corresponded to a larger coefficient of friction.

## 1. Introduction

Fretting occurs when the amplitude of oscillation is low in a sliding motion. A higher amplitude corresponds to reciprocating sliding [1]. Different sliding regimes can be identified, dependent on the relative displacement and the normal load: stick, partial slip, and gross slip [2,3].

Lubrication is one of approaches to prevent fretting damage. It was found that the palliative effect of lubrication against fretting wear was significant in the gross slip regime, compared to dry friction conditions [4]. The fretting performance was investigated in the oil lubrication regime for a large range of amplitude of oscillations from 2 to 400 mm; in the gross slip regime, a lower coefficient of friction was achieved compared to the dry fretting condition [5]. However, in the partial slip regime, the coefficient of friction under oil lubrication was larger than that under dry friction conditions [5,6]. At a higher amplitude or at the final stage of fretting, a transition from high to low wear volume was observed for oil lubrication [5,7,8]. Solid lubricants are the most efficient in the partial slip condition, while grease and oil, especially the latter, are more suitable in the gross slip regime [9]. The fretting behavior in lubricated conditions is dependent on parameters influencing the ability of the lubricant to penetrate the contact; to protect the contact [10], a lubricant can eliminate oxygen from the contact. Larger displacement amplitudes offer an increased lubricant penetration to the contact zone.

Oil temperature affects the fretting behavior. Generally, in the condition of starved lubrication, a high temperature reduces the mechanical strength of the lubricant film and the oil viscosity, leading to increased friction and wear. A high temperature also promotes tribochemical reactions on the sliding surfaces. These reactions can be beneficial or detrimental [11]. An increase in temperature causes a reduction in the lubricant viscosity, which helps the lubricant penetrate the contact, leading to wear limitation. An increase in temperature also may weaken the support offered by the lubricant. For contacts which exhibited sufficient lubricant penetration, the wear rates increased as the temperature increased from ambient to 150 °C [10]. Narayanan et al. [12] studied the effect of temperature on the lubricated fretting wear of tin-plated copper contacts; they also observed a higher wear rate at higher temperatures, however they attributed this behavior to the thermal softening of the tin coating.

Generally, in the fretting regime, due to very small amplitude of oscillation in reciprocating motion, the lubricant penetration to the contact zone is poor [13]. Low-viscosity oils were more effective at suppressing friction and wear than high-viscosity lubricants due to the easier penetration to the contact area [5,6]. The effects of low-viscosity oils on the fretting performance of the as-drawn wire were examined. Oil bath lubrication suppresses effectively the friction and wear [14]. The boundary lubrication properties of the oil had a substantial effect on reducing wear and friction [7,15]. Neyman [16] reported that the effect of the boundary lubrication properties on the fretting wear was more important than the influence of oil viscosity.

In lubricated fretting, a rough contact surface may help to maintain oil, so friction and wear can be reduced. In experiments reported by Sato et al. [7], there were low wear levels for the rough surface and high wear levels for the smooth texture. A significant reduction in fretting wear was achieved by grooving the surface; the beneficial effect was obtained when the spacing between grooves was small [17]. Kubiak and Mathia [18] analyzed the effect of surface texture on lubricated fretting. It was found that the initial surface roughness had a strong effect on the coefficient of friction at the transition between partial slip and gross slip; this coefficient was lower for a rougher surface. Jibiki et al. studied the effect of surface texturing on lubricated fretting [19,20]. The gross-grooved type of texturing was the most efficient for the acceleration of the running-in period [20].

The authors of this paper have not found publications considering the influence of the surface topography on friction and wear in lubricated fretting at different temperatures and normal loads. The present work attempts to fill this gap. Perhaps the normal load affects the lubricant penetration to the contact area and the surface texture effect on the friction and wear would be different at various contact pressures.

## 2. Experimental Details

**Figure 1:**
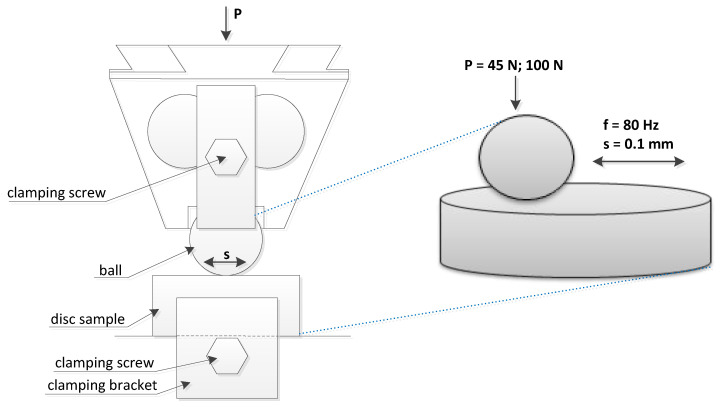
Scheme of the tribotester.

Experiments were conducted using an Optimol SRV5 tester in lubricated friction conditions. Steel balls from 100Cr6 material of 60 HRC hardness were placed in contact with 42CrMo4 steel discs of 47 HRC hardness. Figure 1 shows a scheme of the ball-on-disc tribotester.

Tests were carried out in lubricated friction conditions. Before each test, one drop of lubricant was supplied to the contact zone. SAE 40 SUPEROL (viscosity index: 94; kinematic viscosity: 110 mm^2^/s at 40 °C and 15.1 mm^2^/s at 100 °C) was used as a lubricant. It contains refined, solvent dewaxed and hydrorefined oil distillates received from crude oil and a package of antioxidation and anticorrosion additives. This oil provides a good lubrication of four-stroke diesel and petrol engines in vehicles and machines operated under moderate conditions. It is recommended for engines without air turbochargers. The ball diameter was 10 mm, the stroke was 0.1 mm, and the frequency was set to 80 Hz. Tests were performed at 25–40% of the relative humidity and the number of cycles was typically set to 72,000 (the test duration was 15 min). In addition, for the load of 45 N and at the temperature of 90 °C, test B1 was carried out for larger number of cycles (288,000, the test duration was 1 h). During each test, the normal load was kept constant. Before tests, specimens were cleaned in acetone. Table 1 presents the experimental conditions.

The Hertzian contact pressures for smaller and larger normal loads were 1655 and 2160 MPa, while the elastic contact diameters were 0.228 and 0.297 mm, respectively. For comparison, dry friction tests were conducted at the same conditions as tests A and C.

Tangential fretting in the ball-on-disc contact was selected because this configuration is the most commonly used in experiments [4,5,9,11,17,18,19,20], therefore the results of the present tests can be compared with the results of other investigations. A steel-steel contact was typically studied [4,5,6,9,11,17,18,20], while a bearing steel was frequently used as a ball material [4,9,11,20]. A palliative effect of lubrication against fretting wear was considered. Therefore, the hardness of the disc was lower than the hardness of the ball because, for a hardness difference, the wear is large in dry fretting [21]. Since liquid lubricants are efficient in gross slip fretting, the operating conditions were selected to obtain the gross sliding regime. The SUPEROL mineral oil was chosen because it contains a small number of additives. In addition, it was shown in the previous research of the authors of this paper that the surface texture effect on dry gross fretting was substantial at the normal load of 45 N [22,23], therefore this load was selected. The greater load was at least twice high as the lower load. The fretting tests were conducted in temperatures similar to those for which kinematic viscosities are given.

The temperature of the fretting couple was controlled by a resistance thermocouple located under a table with a tested disc. This table was placed in an isolated chamber. The normal force was applied to the upper specimen (ball) holder by a compression spring system. The range of normal loads was 0–2000 N ± 1 N. A displacement sensor was located near coils causing an oscillatory motion.

**Figure 2:**
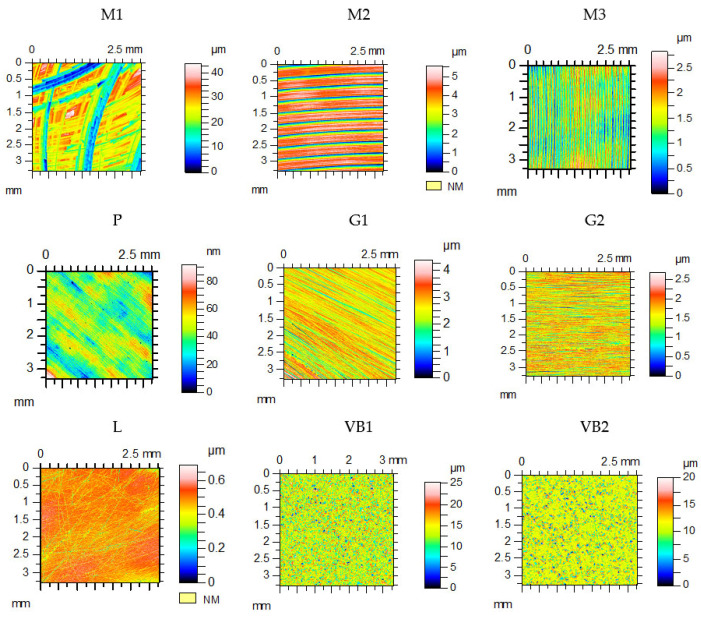
Contour plots of disc surfaces.

The Ra parameter of spheres was 0.15 µm. The discs were machined by vapor blasting (VB), grinding (G), milling (M), lapping (L), and polishing (P). The disc surfaces after machining and wear were measured by a Talysurf CCI Lite optical profilometer (white light interferometer). The measured area was 3.29 × 3.29 mm^2^. Before the computation of the parameters, the surfaces were leveled. Table 2 lists the values of selected surface topography parameters of discs after machining: the standard deviation of height Sq, skewness Ssk, kurtosis Sku, autocorrelation length Sal, texture aspect ratio Str, and rms. slope Sdq [24]. Figure 2 shows the contour plots of disc samples.

The textures of the disc samples after vapor blasting VB1 and VB2 were isotropic (Str parameter had values in the range 0.86–0.87). The values of the Sdq parameter of these topographies were the largest. However, the surface topographies of the disc samples after finish milling M2 and M3 and grinding G1 and G2 were anisotropic (one-directional). From among them, the M3 specimen was characterized by the biggest amplitude. The disc after rough milling M1 was characterized by the biggest height from all tested samples. The Rms. heights of the polished P and lapped L disc specimens were the lowest. The textures of the milled surfaces had a deterministic (after cutting) character, while the textures of the ground, vapor blasted, and lapped surfaces had a random character (after abrasive processes).

**Figure 3:**
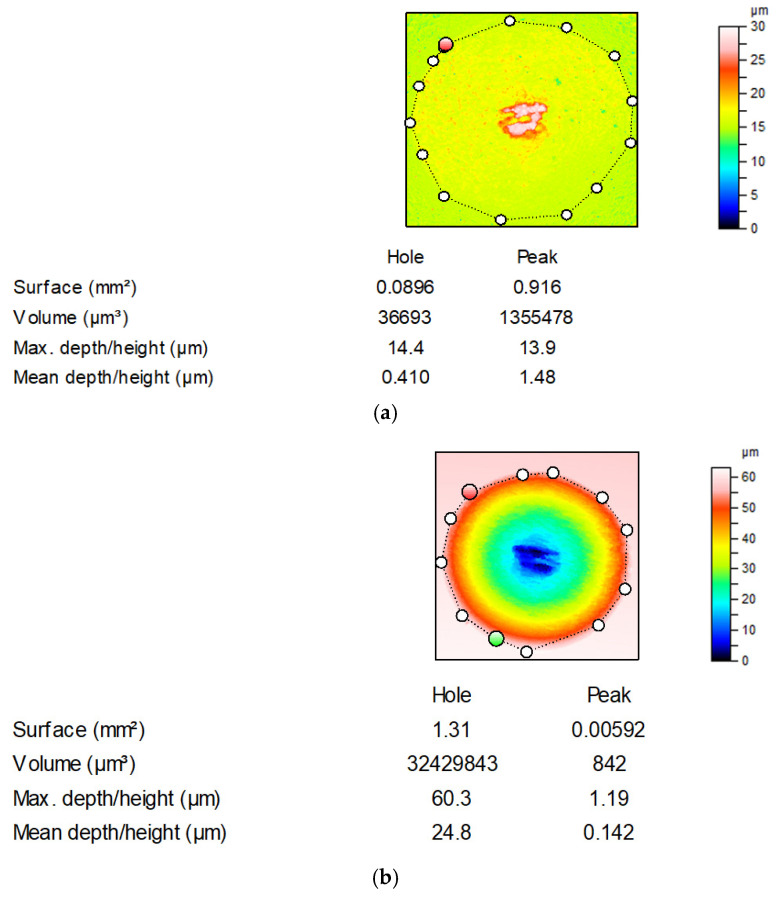
Example of the estimation of the total wear volume of the tribological system: wear of ball: (V_ball_−) = 36,693 µm^3^, (V_ball_+) = 1,355,478 µm^3^, V_ball_ = −1,318,785 µm^3^ (**a**); wear of disc: (V_disc_−) = 32,429,843 µm^3^, (V_disc_+) = 842 µm^3^, V_disc_ = 32,429,001 µm^3^ (**b**); total volumetric wear: V_total_ = 31,110,216 µm^3^.

Anisotropic milled and ground surfaces were positioned perpendicularly to the main texture direction (lay). Prior to the estimation of the wear level of the ball, its curvature was removed by a sphere. The total volumetric wear was computed as Vtotal = (Vdisc−) − (Vdisc+) + (Vball−) − (Vball+), Vdisc = (Vdisc−) − (Vdisc+), Vball = (Vball−) − (Vball+); volumes (Vdisc+) and (Vball+) were transferred materials or buildups and volumes (Vdisc−) and (Vball−) were lost materials [10]. They were calculated with the use of TalyMap software; (Vdisc+) or (Vball+) is the volume of the peak and (Vdisc−) or (Vball−) is the volume of the hole. Figure 3 shows the example of wear volume estimation. The white light interferometer was recently calibrated. After the measurement of the surface RMS, it was qualified as capable. Because of its vertical resolution of 0.01 nm, small wear volumes could be correctly determined. A special procedure was used to minimize the effect of errors on the wear volume estimation [25]. The volume of the hole was computed between the bottom of the hole and the least square plane. This plane was calculated for the surface points outside the hole.

Each set of test conditions was repeated at least three times.

## 3. Results

The average coefficient of friction was computed after removing the initial fluctuations after 50 s (COF_50–900_) and the final friction coefficient was calculated after 600 s (last 5 min—COF_600–900_). A scatter of the average coefficient of friction is the difference between the maximum and minimum values of the average coefficients of friction for repetitions of the tests. A scatter of the final coefficient of friction and a scatter of the total volumetric wear were calculated by similar way.

**Figure 4:**
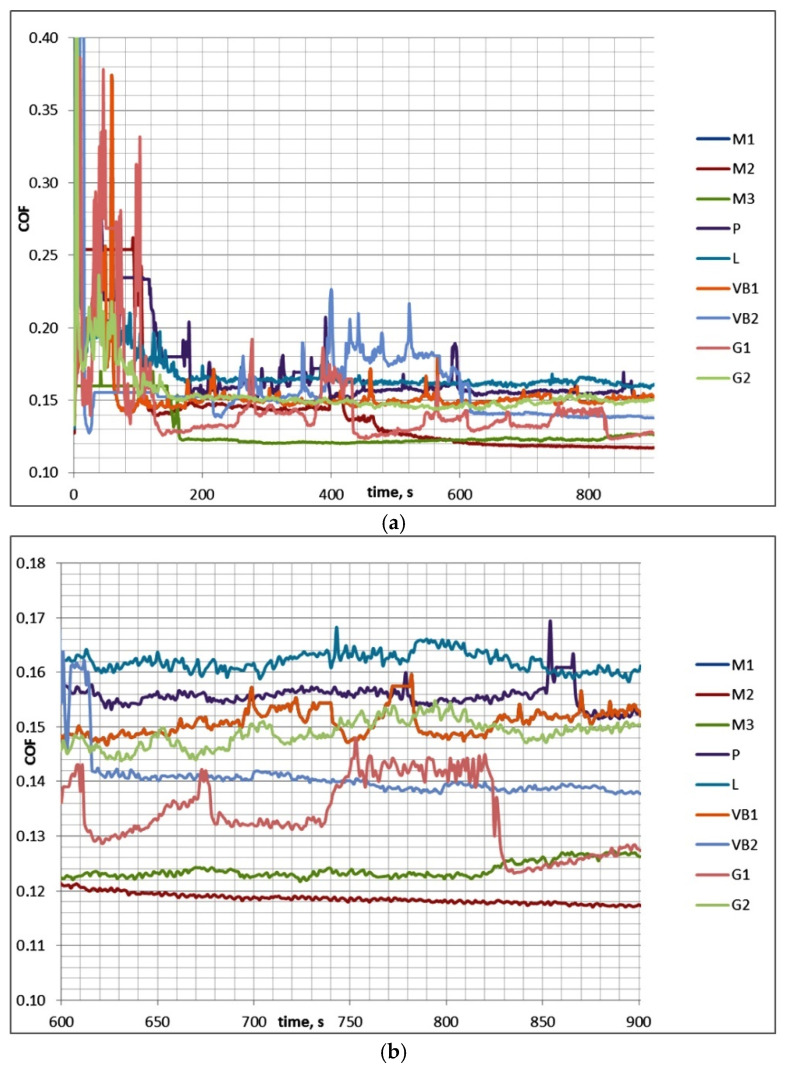
Friction coefficients for the whole test A (**a**) and for its last 5 min (**b**).

Table 3 presents the results of investigations for test A (mean values), while Figure 4 shows examples of curves presenting the friction coefficient versus time.

In test A after initial fluctuations, the coefficient of friction decreased and reached a stable value. Therefore, COF_50–900_ was higher than COF_600–900_. The scatter of the friction coefficient for the same assembly in the final test part COF_600–900_ was typically smaller than 0.01, which was lower than the scatter of the coefficient of friction COF_50–900_, which was 0.02. The decreases in the coefficients of friction in time were in most cases marginal. Typically, the wear level of discs was higher than that of balls, and buildups or material transfers on ball surfaces were observed (in contrast to the assemblies with the samples M2 and VB2). The scatters of the total volumetric wear were smaller than 80,000 µm^3^. The highest final coefficient of friction was 1.4 times larger than the smallest one; the corresponding ratios of the total wear volumes of sliding pairs were much higher.

**Figure 5:**
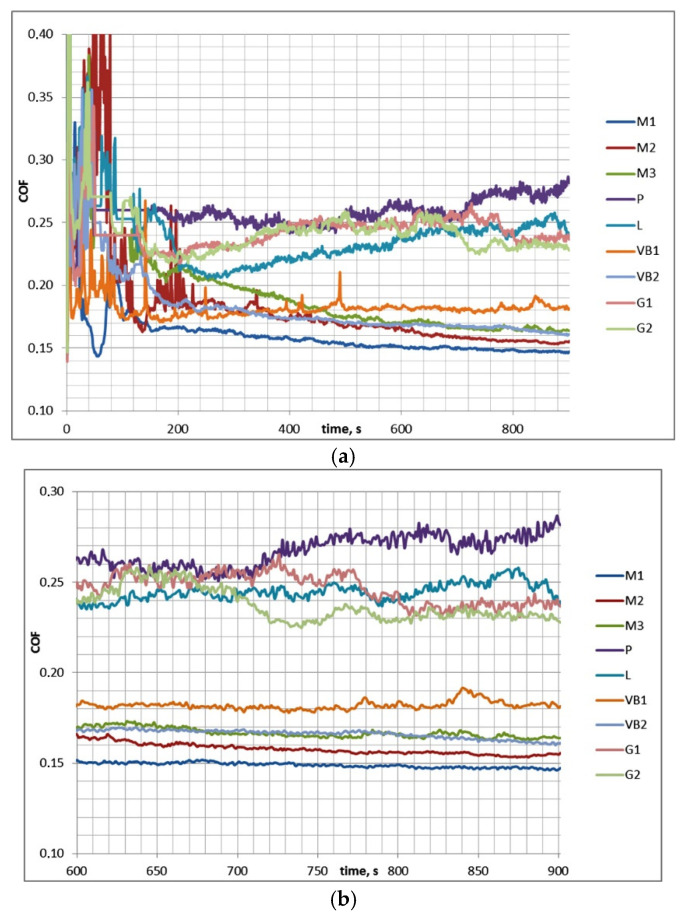
Friction coefficients for whole test B (**a**) and for its last 5 min (**b**).

Table 4 and Figure 5 show the results of test B.

In test B, the coefficients of friction after the initial fluctuations behaved differently after 3 min of test runs for two sets of assemblies: they increased for sliding pairs with the ground G1 and G2 disc samples and with disc specimens after polishing P and lapping L; however, for the assemblies with the other samples (after milling and vapor blasting), they decreased. The scatters of the coefficient of friction (both COF_50–900_ and COF_600–900_) for the same assemblies were smaller than 0.018. The maximum final coefficient of friction COF_600–900_ was nearly twice as high as the minimum one. In most cases, the negative wear values of ball samples caused by material transfers or build-ups were noticed, but the opposite situation took place for assemblies with rough disc samples after vapor blasting VB1 and VB2. The scatters of the total wear volumes for different sliding pairs were between 40,000 and 180,000 µm^3^ (larger variations corresponded to higher wear levels). The test for the same sliding pair was repeated at least three times and the scatter was calculated based on the different wear volumes given for this specific sliding pair.

**Figure 6:**
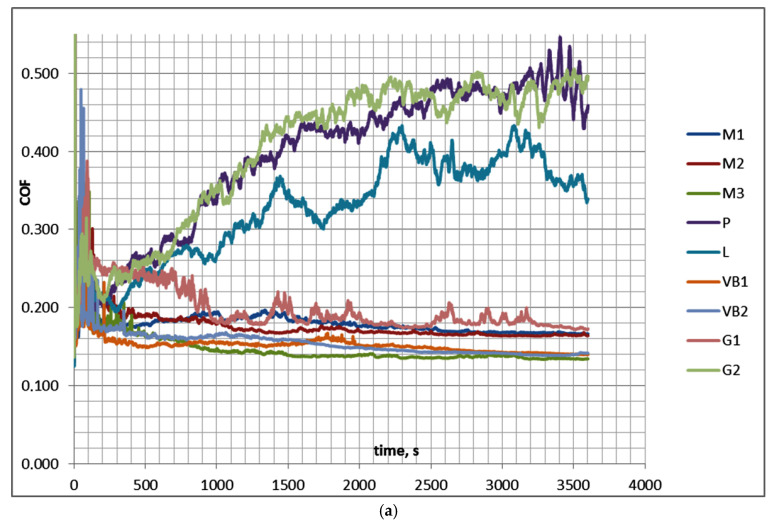

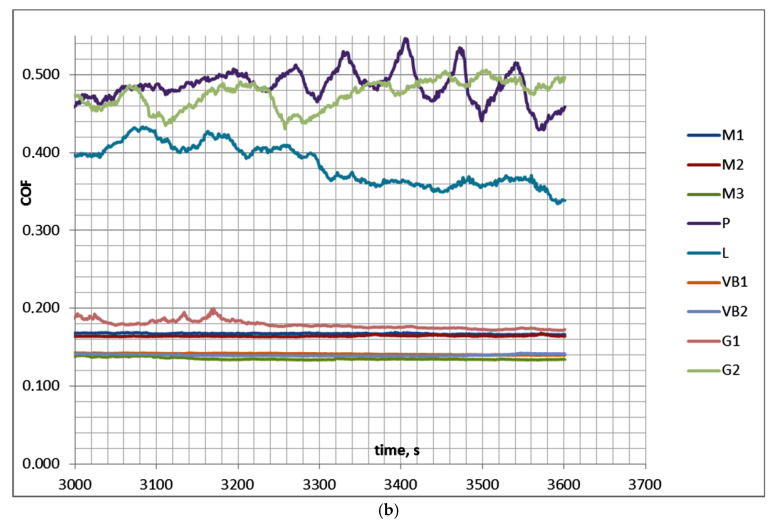
Friction coefficients for the whole test B1 (**a**) and for its last 10 min (**b**).

Because in test B a large number of test runs had not reached a steady state, test B1 was conducted under the same operating conditions as test B, but the number of cycles was increased to 288,000 (test duration was 1 h). Table 5 and Figure 6 show the results of test B1. The average value of the coefficient of friction was assessed between 50 and 3600 s (COF_50–3600_), while the final friction coefficient was evaluated between 3000 and 3600 s (last 10 min—COF_3000–3600_).

In test B1, similarly to test B, after initial fluctuations during the first 10 min the coefficient of friction behaved differently for sliding pairs with disc samples P, L, and G2 and for other assemblies. For the first group of sliding pairs with the disc surfaces of the smallest height (P, L, and G2), the coefficient of friction increased as the test progressed and obtained a stable value after approximately 2100 s. These surfaces led to unstable friction due to the low roughness height. In this case, the average coefficient of friction COF_50–3600_ was smaller than the final friction coefficient COF_3000–3600_. When other assemblies were tested, the coefficient of friction decreased and reached a stable value after 1000 s of the test run. In this case, COF_50–900_ was higher than COF_3000–3600_. Within this group, the disc sample G1 yielded the largest coefficient of friction, up to 700 s, and then the friction coefficient decreased. The scatters of the average and final coefficients of friction for the same sliding pairs were smaller than 0.02. The maximum final coefficient of friction COF_3000–3600_ was more than 3.5 times higher than the smallest one. For all tested assemblies, the wear level of the disc was higher than that of the balls, and in most cases negative wear values of the ball samples caused by material transfers or build-ups were observed; the sliding pair with the VB1 disc sample was the exception. Due to an increase in the test duration, both the volumetric wear and its variation increased. The scatters of the total volumetric wear levels, due to repetitions of the tests, were between 200,000 and 1,200,000 µm^3^ (larger variations corresponded to higher wear levels).

**Figure 7:**
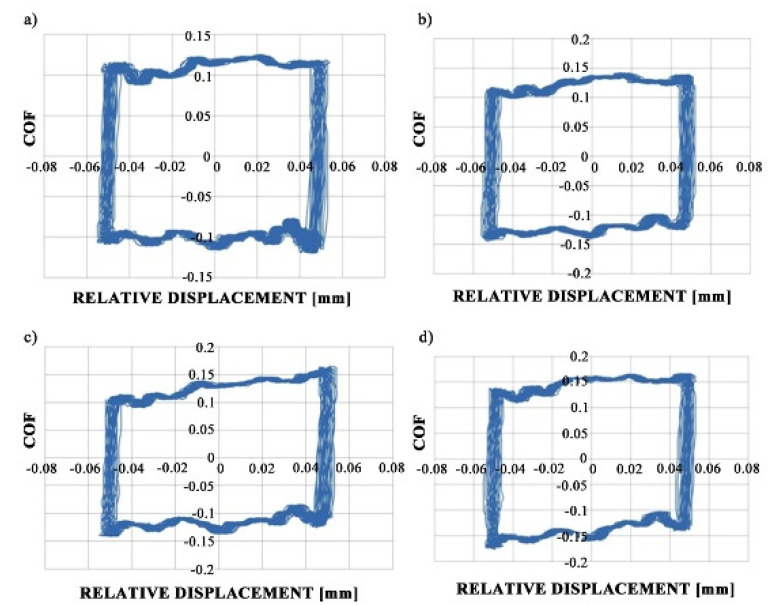
Fretting loops for assemblies with M1 (**a**,**c**) and VB1 discs (**b**,**d**) during tests A (**a**,**b**) and B (**c**,**d**).

Figure 7 shows fretting loops for sliding pairs containing samples M1 and VB1 during tests A and B; these loops were obtained after 10 min. An increase in temperature did not affect the shapes of the fretting loops but caused the growth of the friction coefficient.

**Figure 8:**
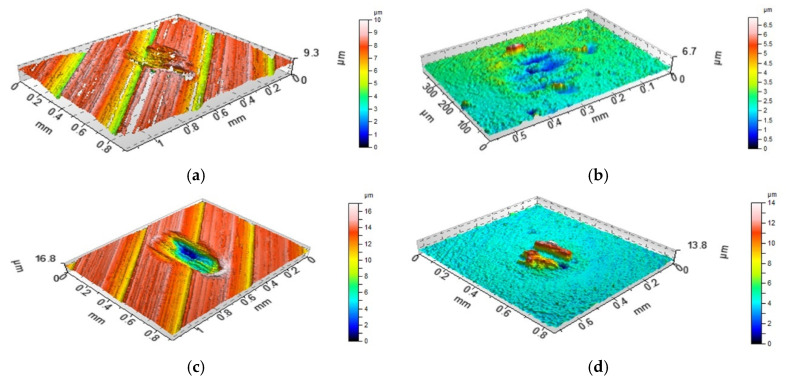
Isometric views of disc M1 (**a**,**c**) and co-acted balls (**b**,**d**) after tests A (**a**,**b**) and B (**c**,**d**).

**Figure 9:**
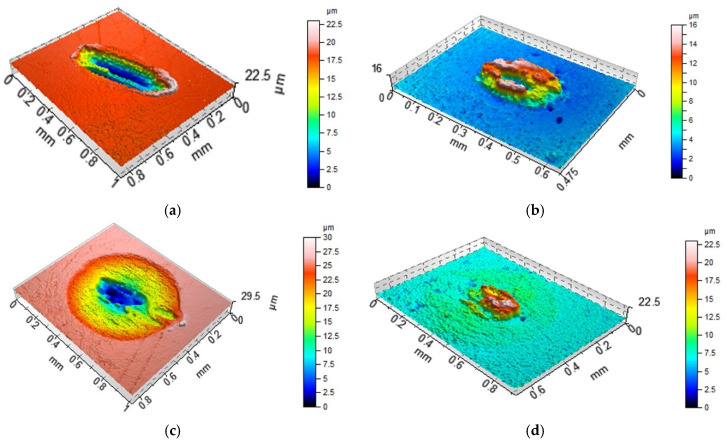
Isometric views of disc L (**a**,**c**) and co-acted balls (**b**,**d**) after tests A (**a**,**b**) and B (**c**,**d**).

**Figure 10:**
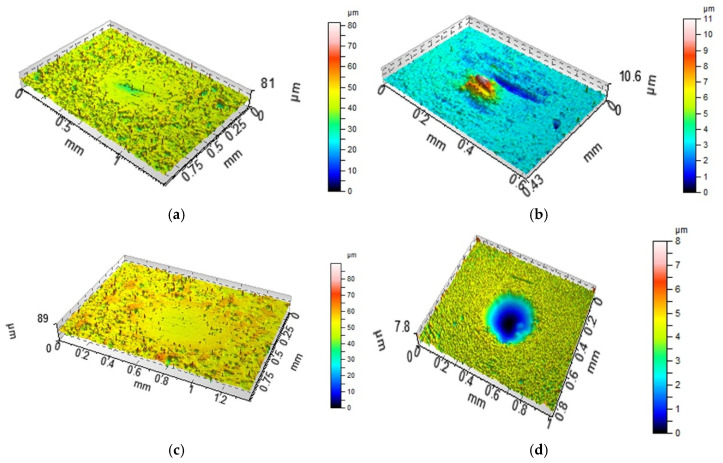
Isometric views of disc VB1 (**a**,**c**) and co-acted balls (**b**,**d**) after tests A (**a**,**b**) and B (**c**,**d**).

Figure 8, Figure 9 and Figure 10 present the contour plots of the selected discs and balls after tests A and B. For assemblies with discs M1 and L (Figure 8 and Figure 9), buildups or material transfers were visible on the ball samples; the wear levels of the discs were higher at elevated temperature. Material loss was observed for the disc surface VB1, while wear and material transfer were visible on the co-acted ball at a lower temperature. At an elevated temperature, only the material loss occurred on the ball surface (Figure 10).

**Figure 11:**
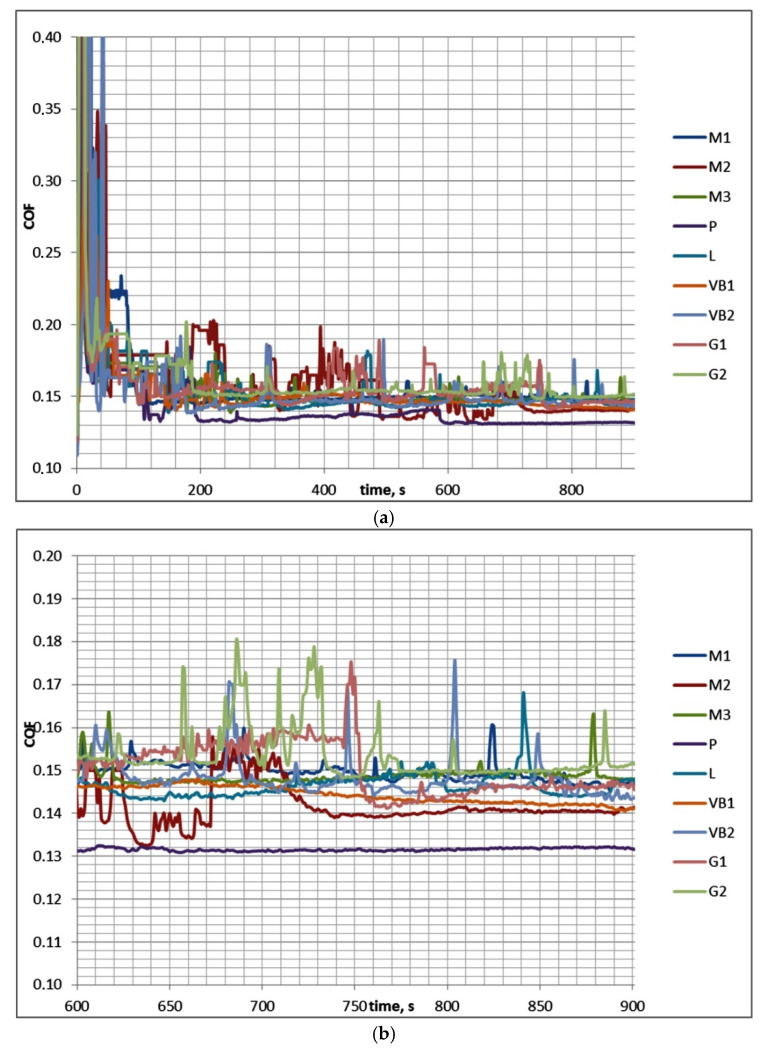
Friction coefficients for whole test C (**a**) and for its last 5 min (**b**).

Table 6 and Figure 11 present the results of test C.

The coefficient of friction in test C after the initial growth decreased and reached a stable value after the initial 2 min. Therefore, COF_50–900_ was marginally higher than COF_600–900_. However, in the final part of the test, typically fluctuations in the coefficient of friction were observed, except for sliding pairs with the P, M2, and VB1 samples. The smallest final coefficient of friction COF_600–900_ was noticed for the sliding pair with the polished disc L (0.133); for other sliding pairs, the final coefficients of friction COF_600–900_ were similar (0.144–0.154). Similar to test A, the scatter of the coefficient of friction for the same assembly in the final test part COF_600–900_ was smaller than 0.01, which was lower than the scatter of the average coefficient of friction COF_50–900_, which was smaller than 0.02. In all analyzed cases, material losses were observed on the disc surfaces, while material transfers or buildups were seen on ball samples. The scatters of total volumetric wear levels were typically smaller than 80,000 µm^3^.

**Figure 12:**
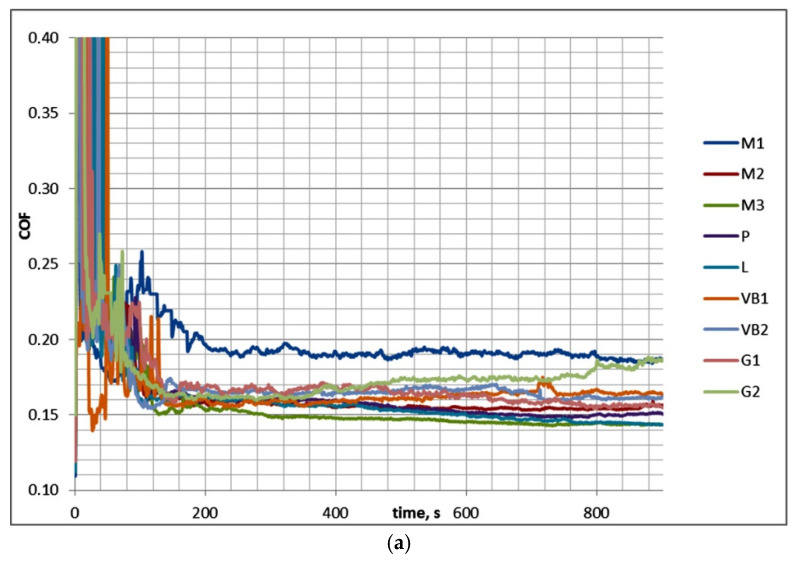

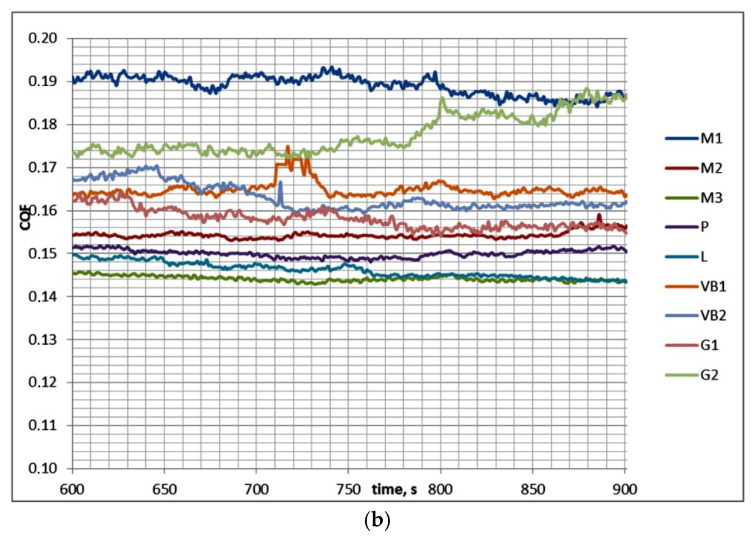
Friction coefficients for the whole of test D (**a**) and for its last 5 min (**b**).

Table 7 presents the results of investigations of tests D, while Figure 12 shows the values of the friction coefficient versus time.

In test D, COF_50–900_ was higher than COF_600–900_; the changes were marginal for sliding pairs with discs M1, VB1, VB2, and G2. Stable values of the friction coefficient were obtained after 200 s. An increase in temperature led to smaller fluctuations in the coefficient of friction after its stabilization. In the final parts of the tests, the coefficients of friction were typically constant; the sliding pair with G2 disc was the exception. The lowest friction coefficient was obtained for the assembly with the smoothest disc surface P and the largest friction coefficient was received for the sliding pair with the disc surface M1 of the biggest height. The maximum COF_600–900_ was about 1.4 times higher than the minimum one. In test D, the scatters of the coefficients of friction for the same assemblies were marginally smaller than those in test C. Similar to test C, material losses were observed on the disc samples, while material transfer or build-ups were observed on balls. The scatters of the total volumetric wear volumes were typically smaller than 150,000 µm^3^.

**Figure 13:**
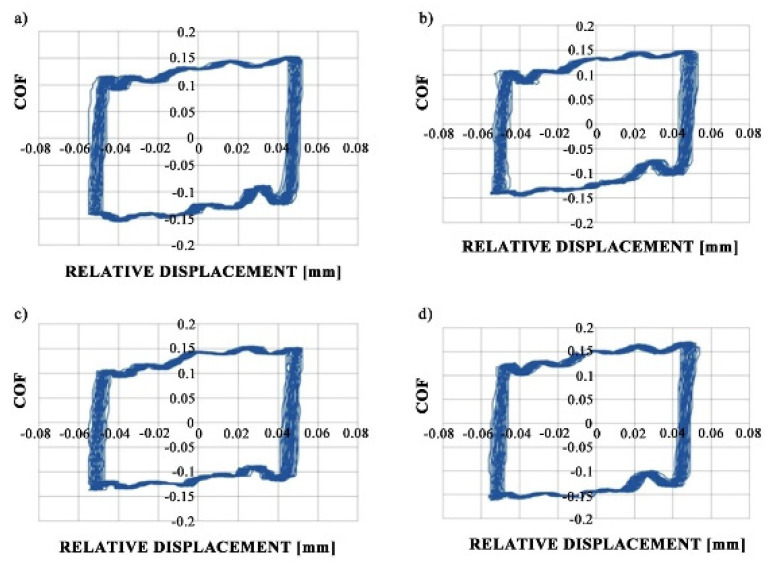
Fretting loops for assemblies with M3 (**a**,**c**) and VB1 discs (**b**,**d**) during tests C (**a**,**b**) and D (**c**,**d**).

Figure 13 presents the fretting loops for sliding pairs containing samples M3 and VB1 during tests C and D. Similar to tests A and B, an increase in temperature did not affect the shape of the fretting loop; however, it caused an increase in the coefficient of friction.

**Figure 14:**
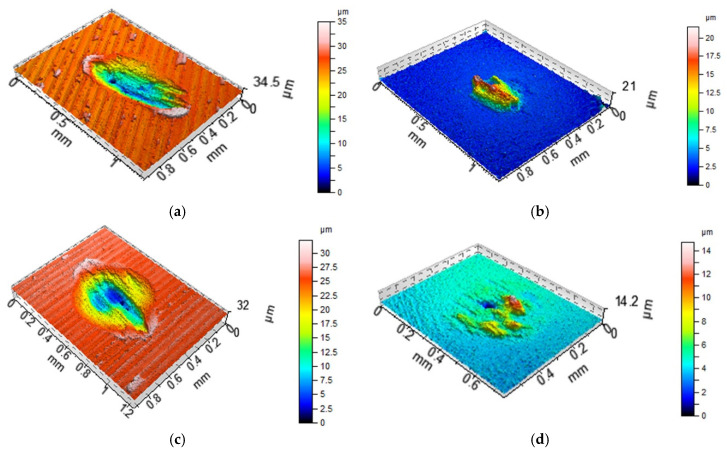
Isometric views of disc M3 (**a**,**c**) and co-acted balls (**b**,**d**) after tests C (**a**,**b**) and D (**c**,**d**).

**Figure 15:**
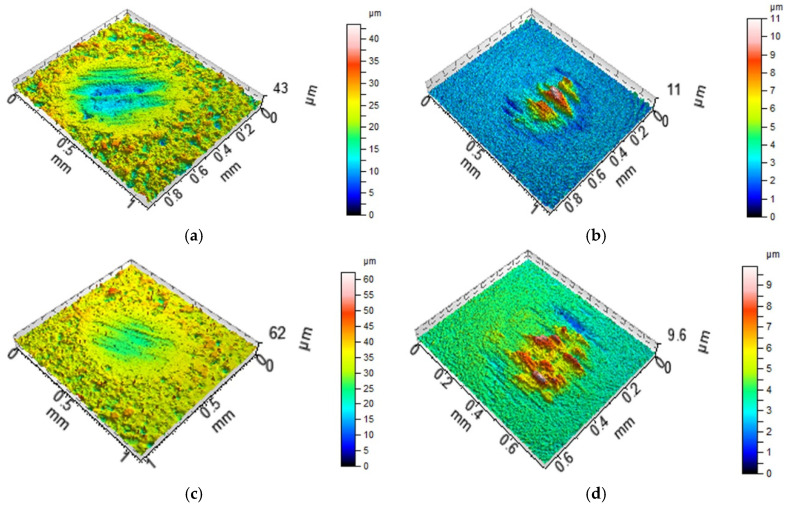
Isometric views of disc VB1 (**a**,**c**) and co-acted balls (**b**,**d**) after tests C (**a**,**b**) and D (**c**,**d**).

Figure 14 and Figure 15 show the contour plots of selected discs and balls after tests C and D. For both assemblies, wear scars on the disc surfaces and material transfers or build-ups on the ball surfaces were observed.

In dry fretting conditions, both coefficients of friction and wear levels were much higher than those obtained in lubricated fretting. The shapes of curves showing the coefficient of friction versus time were also different. When the normal load was smaller (45 N), the coefficient of friction after the abrupt initial growth increased and obtained a stable value after 10 min of test. Therefore, in contrast to lubricating fretting, COF_50–900_ (0.88–0.97) was smaller than COF_600–900_ (0.98–1.07). For larger load (100 N) the coefficient of friction after early sharp growth increased gradually and was stabilized after 5 min of the test run with possible marginal further increase. Similar to dry test conducted at smaller normal load, COF_50–900_ (0.83–0.93) was smaller than COF_600–900_ (0.9–1); however, at higher load the differences between final and mean coefficients of friction were smaller.

In dry fretting, an increase in the normal load led to smaller coefficient of friction. The smallest resistances to motion were obtained for sliding pairs with finish milled disc samples M2 and M3. Wear levels of balls were much higher than those of discs, this behavior was caused by oxidized debris of high hardness embedding into softer disc materials [21]; a similar performance was found in the other work [22]. Similar to the coefficient of friction, the wear level decreased as the normal load increased. For smaller loads, the mean volumetric wear value was 12,925,204 µm^3^, while for higher load the average wear volume was 10,117,580 µm^3^. When the load was higher, total wear was proportional to the final coefficient of friction COF_600–900_ (the coefficient of determination was 0.85). We found from the analysis of fretting loops that gross slip regime occurred. An increase in the normal load caused a decrease in the slip index [26,27], typically from about 3 to about 1.5.

## 4. Discussion

From the friction data, it can be seen that there were severe adhesive interactions within the first 200 s. During this phase, material transfer and plastic deformation took place and probably chemical products were produced. These abrupt changes in the coefficient of friction are related to instable lubrication (combination of dry and lubricated contact). The standard deviations of the coefficient of friction were up to 0.1 in this period.

The experiments were carried out in the gross slip regime, oil penetration capacity controlled the fretting behavior, and a lower coefficient of friction and lower wear were achieved compared to fretting tests under dry conditions. Similar results were obtained in other works [4,5,8]. However, in lubricated fretting wear levels of discs were larger than those of balls, in contrast to dry fretting conditions.

When the normal load was smaller, an increase in temperature led to an increase in the coefficient of friction, both average and final, about 1.4 times on average. Under these conditions, the total volumetric wear of the tribological system increased more than three times due to the temperature growth, which is evident in Figure 8 and Figure 9 (both areas and depths of wear scars increased). The temperature increase weakened the support offered by the lubricant that penetrated the contact. An increase in wear at elevated temperatures was also found in works [12,19]. However, different changes were observed when larger normal load was applied. In these conditions, due to temperature increase, the coefficient of friction increased, while total wear decreased marginally. For both loads increases in temperature led to bigger stability of friction. An increase in load caused marginal changes in the coefficient of friction and an increase in wear at smaller temperature. At higher temperatures, an increase in load caused decreases in the coefficient of friction and wear.

**Figure 16:**
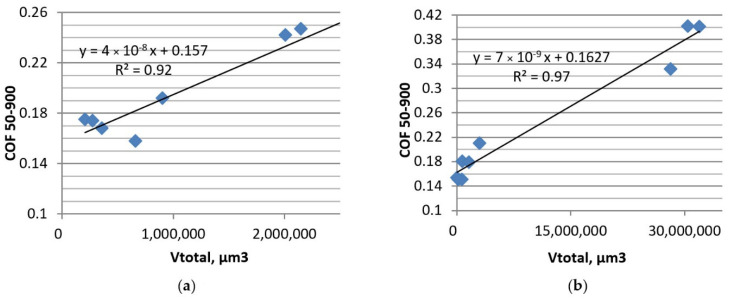
Average coefficients of friction versus volumetric wear of the tribological system in tests: B (**a**) and B1 (**b**).

In most cases, the volumetric wear levels were proportional to the coefficients of friction, except for test C. This dependence was the strongest for test B and test B1 (Figure 16), when the ratios of the largest to the lowest coefficients of friction and wear levels were the highest. Shima et al. [6] also observed a strong correlation between the coefficient of friction and the amount of wear.

**Figure 17:**
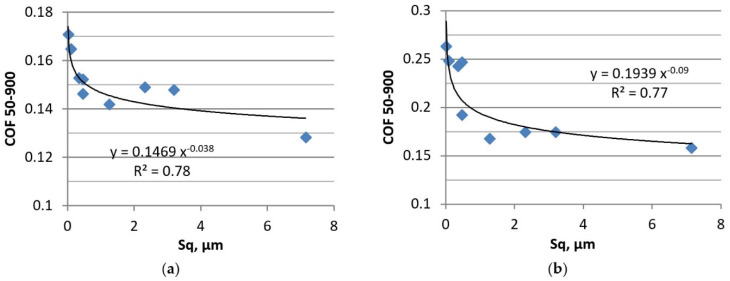
Dependence between the surface height of disc and the coefficient of friction in tests A (**a**) and B (**b**).

The different reactions of the tribological system to temperature increase in tests A and B compared to tests C and D were probably related to the various effects of disc surface topography on the friction and wear. The opinion exists that, since rough contact surface helped to retain oil, the friction and wear should be decreased. This assumption was confirmed in experimental research [7,17]. In the present study, the beneficial effect of rough surfaces on the tribological performance in lubricated fretting was observed only for smaller applied normal loads. This effect was more evident at higher temperatures, because low-viscosity oil more easily penetrated the contact area than high-viscosity lubricant. Figure 17 presents the dependencies between the values of the Sq parameter of disc surfaces and the average coefficients of friction in tests A and B.

**Figure 18:**
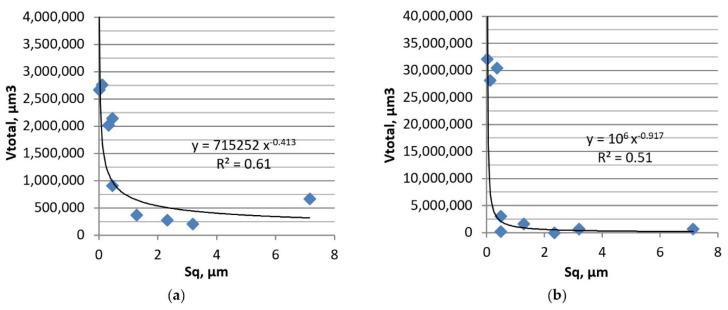
Dependence between the surface height of the disc and the total volumetric wear of the tribological system in tests B (**a**) and B1 (**b**).

Figure 18 shows the dependence between the values of the Sq parameter of disc surfaces and the total volumetric wear levels of the tribological systems in tests B and B1. Rougher surfaces led to lower wear volumes.

In test A, the smallest coefficient of friction and volumetric wear was obtained for milled samples, while the highest was obtained for the smoothest samples after polishing and lapping. The frictional resistance and wear of sliding pairs containing discs after vapor blasting and grinding were similar.

An increase in temperature (test B) led to the smallest wear volumes of assemblies with vapor blasted disc samples followed by those with milled, ground, lapped and polished samples. The wear levels of systems with polished or lapped discs were about 10 times higher than those of assemblies with disc samples after vapor blasting. It is interesting that material losses were not observed on the samples after vapor blasting, only on the co-acted balls. The samples after milling and vapor blasting led to low friction, while the samples after grinding, polishing, and lapping led to high friction. Good frictional properties of sliding pairs with disc samples after finish milling M2 and M3 are interesting—the beneficial functional behaviors of similar textures were confirmed in dry fretting tests [22]. These one-directional anisotropic surfaces were formed by a cutting process and had a deterministic character; the peaks were milder than those formed by grinding. In dry sliding conditions, a low coefficient of friction of assembly containing milled surfaces was achieved when the sliding direction was perpendicular to the main texture direction (lay). This beneficial frictional performance was probably related to the formation of the oxide layer and its load-carrying capacity. One-directional character of the milled samples was also probably the reason for the low friction in the lubricated regime.

An increase in the test duration from 15 to 60 min (test B1) caused a similar tribological performance of sliding pairs compared to test B. The behavior of the assembly with the disc G1 was the exception—for a shorter test duration (test B), the coefficient of friction was comparatively high, while for a larger number of cycles it decreased.

Under smaller load, rough surfaces led to smaller friction and wear than smooth textures, because rough surfaces contained many micro-pockets which can retain lubricant in the contact area. An increase in temperature led to a reduction in the oil viscosity, which encouraged the lubricant to penetrate to the contact area; therefore, the differences between the highest and the smallest coefficients of friction and wear levels increased at elevated temperature.

**Figure 19:**
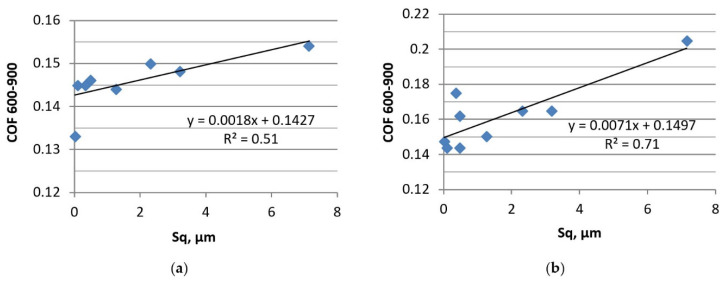
Dependence between the surface height of disc and the coefficient of friction in tests C (**a**) and D (**b**).

Different behavior of the tribological system was obtained when the normal load was higher (Figure 19).

In both tests (C and D), the coefficient of friction was higher for rougher disc surfaces. In test C, the ratio of the highest to the smallest coefficients of friction was comparatively low (1.15), but it increased at elevated temperatures (test D). The ground discs led to the highest volumetric wear in test C. In test D, the smallest coefficient of friction was achieved for sliding pairs with polished, lapped, and finish milled discs, followed by those with ground, vapor blasted, and rough milled samples. Discs after finish milling M2 and M3 led to the smallest wear levels, however discs after grinding and rough milling led to the highest wear levels.

No beneficial effect of an increase in the height of the disc surface on the tribological performance of the sliding pair in lubricated conditions was found under a higher normal load of 100 N. In fretting, owing to very small amplitude of motion, the lubricant penetration into the contact zone is poor. Perhaps at a higher normal load, because of the large contact pressure, the lubricant could not penetrate to the contact area, so the rough disc could not help to maintain oil. In these conditions, higher amplitudes of surfaces resulted in higher coefficients of friction. When surfaces are in sliding contact in lubricated conditions, the roughness has a significant influence on the friction; due to an increase in the roughness height, the friction increases. This phenomenon can be explained by the negligible adhesion effects in lubricated sliding. Similar results were obtained in other experimental works [28,29,30,31].

An increase in temperature led to smaller fluctuations in the friction force.

## 5. Conclusions

The authors of this paper found that, in the gross slip fretting regime, the coefficient of friction under oil lubrication was smaller than that under dry conditions. The wear of the ball was lower than the wear of the disc in lubricated fretting. Under these conditions, an increase in temperature from 30 to 900 °C led to more stable friction force.

The amplitude of the surface texture had significant effects on the friction and wear in lubricated fretting. These effects are more substantial at a higher temperature of 900 °C and depend on the contact pressure.

When a smaller normal load of 45 N was applied, the coefficient of friction was lower for greater disc roughness height for temperatures of 30 and 900 °C. An increase in temperature led to greater friction and wear. At a higher temperature of 900 °C, the wear volumes were larger for the lower roughness height in lubricated fretting.

In lubricated fretting for a larger normal load of 100 N, higher roughness corresponded to a larger coefficient of friction. An increase in temperature from 30 to 900 °C caused higher coefficients of friction and negligible changes in the wear levels.

Typically, the wear level was proportional to the coefficient of friction in lubricated fretting.

## Figures and Tables

**Table 1:** Experimental conditions under lubrication.

Test Designation	Temperature, °C	Normal Load, N	Number of Cycles
A	30	45	72,000
B	90	45	72,000
B1	90	45	288,000
C	30	100	72,000
D	90	100	72,000

**Table 2:** Parameters of the tested disc surfaces.

Surface Type	Sq, µm	Ssk	Sku	Sal, mm	Str	Sdq
M1	7.15	−0.465	2.81	0.187	0.282	0.288
M2	1.27	−0.8	3.19	0.059	0.04	0.0924
M3	0.48	0.38	2.87	0.025	0.0154	0.074
P	0.012	−0.9	5.6	0.194	0.311	0.0029
L	0.11	−0.84	4.4	0.014	0.162	0.021
VB1	3.2	−0.32	4.04	0.021	0.864	0.58
VB2	2.32	−0.38	4.6	0.019	0.877	0.43
G1	0.48	−0.6	3.9	0.016	0.035	0.116
G2	0.35	−0.53	3.6	0.009	0.0185	0.099

**Table 3:** Results of test A (f = 80 Hz, P = 45 N, temperature 30 °C, the number of cycles 72,000).

Disc Sample	COF_50-900_	COF_600-900_	V_disc_, µm^3^	V_ball_, µm^3^	V_total_, µm^3^
M1	0.128	0.121	79,959	−25,505	54,454
M2	0.142	0.12	−10,666	17,892	7226
M3	0.146	0.122	63,927	−59,341	4586
P	0.171	0.17	1,763,156	26,455	1,789,611
L	0.165	0.164	1,099,153	−322,797	776,356
VB1	0.148	0.147	161,656	−18,826	142,830
VB2	0.149	0.148	−55,769	343,937	288,168
G1	0.152	0.143	168,377	−57,743	110,634
G2	0.153	0.151	609,809	−135,498	474,311

**Table 4:** Results of test B (f = 80 Hz, P = 45 N, temperature 90 °C, the number of cycles 72,000).

Disc Sample	COF_50–900_	COF_600–900_	V_disc_, µm^3^	V_ball_, µm^3^	V_total_, µm^3^
M1	0.158	0.147	783,237	−125,680	657,557
M2	0.168	0.161	494,999	−137,850	357,149
M3	0.192	0.172	1,200,777	−297,504	903,273
P	0.263	0.274	3,046,432	−381,643	2,664,789
L	0.248	0.251	3,253,148	−497,719	2,755,429
VB1	0.175	0.175	−61,575	265,154	203,579
VB2	0.174	0.174	−105,387	380,282	274,895
G1	0.247	0.25	2,576,665	−429,696	2,146,969
G2	0.242	0.244	2,460,779	−452,710	2,008,069

**Table 5:** Results of test B1 (f = 80 Hz, P = 45 N, temperature 90 °C, number of cycles 288,000).

Disc Sample	COF_50–3600_	COF_3000–3600_	V_disc_, µm^3^	V_ball_, µm^3^	V_total_, µm^3^
M1	0.181	0.172	1,069,547	−368,870	700,677
M2	0.179	0.162	1,825,580	−255,905	1,569,675
M3	0.151	0.142	411,650	−229,994	181,656
P	0.401	0.493	33,184,984	−1,217,201	31,967,783
L	0.332	0.383	28,983,040	−809,000	28,174,040
VB1	0.151	0.139	629,272	10,433	639,705
VB2	0.154	0.142	450,036	−431,592	18,444
G1	0.21	0.179	3,528,540	−519,526	3,009,014
G2	0.402	0.472	30,863,532	−457,070	30,406,462

**Table 6:** Results of test C (f = 80 Hz, P = 100 N, temperature 30 °C, the number of cycles 72,000).

Disc Sample	COF_50–3600_	COF_3000–3600_	V_disc_, µm^3^	V_ball_, µm^3^	V_total_, µm^3^
M1	0.156	0.154	826,359	−367,861	458,498
M2	0.147	0.144	1,217,615	−335,792	881,823
M3	0.146	0.146	1,519,230	−408,371	1,110,859
P	0.139	0.133	1,549,014	−439,183	1,109,831
L	0.146	0.145	1,752,391	−510,474	1,241,917
VB1	0.154	0.148	1,282,293	−239,160	1,043,133
VB2	0.154	0.15	1,112,101	−405,750	706,351
G1	0.15	0.146	1,970,390	−453,543	1,516,847
G2	0.155	0.145	2,310,626	−644,406	1,666,220

**Table 7:** Results of test D (f = 80 Hz, P = 100 N, temperature 90 °C, the number of cycles 72,000).

Disc Sample	COF_50–900_	COF_600–900_	V_disc_, µm^3^	V_ball_, µm^3^	V_total_, µm^3^
M1	0.210	0.205	2,207,939	−468,375	1,739,564
M2	0.163	0.15	599,301	−236,004	363,297
M3	0.154	0.144	747,812	−240,411	507,401
P	0.156	0.147	1,119,516	−265,246	854,270
L	0.164	0.144	1,372,969	−414,434	958,535
VB1	0.167	0.165	1,014,124	−170,540	843,584
VB2	0.174	0.1645	967,239	−298,618	668,621
G1	0.171	0.162	1,884,768	−611,583	1,273,185
G2	0.176	0.175	2,058,178	−549,595	1,508,583
